# Combining Radiation‐Treated Tumor Vaccines With Mn‐MOF Nanoadjuvants to Amplify Radiation Induced Anti‐Tumor Immune Responses

**DOI:** 10.1002/advs.75860

**Published:** 2026-05-29

**Authors:** Yiyu Wang, Qiqi Qi, Dezhong Li, Jingshi Tang, Weifeng Wang, Zushun Xu, Qi Xu, Fangfang Du, Qianyuan He

**Affiliations:** ^1^ NHC Key Laboratory of Tropical Disease Control Engineering Research Center for Hainan Bio‐Smart Materials and Bio‐Medical Devices Key Laboratory of Hainan Functional Materials and Molecular Imaging School of Life Sciences and Medical Technology Hainan Medical University Haikou Hainan China; ^2^ Key Laboratory of Emergency and Trauma Key Laboratory of Haikou Trauma Key Laboratory of Hainan Trauma and Disaster Rescue Ministry of Education The First Affiliated Hospital Hainan Medical University Haikou China; ^3^ College of Life Sciences Yangtze University Jingzhou Hubei China; ^4^ School of Materials Science and Engineering Hubei University Wuhan Hubei China; ^5^ Hainan Cancer Hospital Affiliated Hospital of Hainan Medical University Haikou Hainan China

**Keywords:** antigen presentation, cancer vaccine, immunotherapy, radiation therapy, tumor microenvironment

## Abstract

Radiotherapy‐induced mutations are critical for neoantigen generation and the initiation of in situ vaccine mediated immune responses; however, their efficacy is severely limited by the immunosuppressive tumor microenvironment. Here, genomic analysis demonstrated that B16F10 tumor cells subjected to in vitro irradiation faithfully recapitulate part of the radiotherapy‐induced mutational landscape observed in subcutaneous tumor models. Based on these findings, a biomimetic cancer vaccine was developed through the integration of RT‐treated tumor cell membranes and manganese‐based metal organic framework nanoadjuvants (Mn@RM). Mn@RM can deliver RT‐induced membrane proteins directly to lymph nodes in order to potentiate the in situ vaccine immune response. Moreover, Mn‐MOF nanoadjuvants enhanced antigen presentation efficiency by nearly two‐fold through activation of the cGAS‐STING pathway in dendritic cells, while Mn@RM further optimized the lymph node immune microenvironment to promote robust anti‐tumor immunity. When combined with radiotherapy and PD‐1 immune checkpoint blockade, Mn@RM demonstrates excellent synergistic tumor treatment effect. Overall, this study offers a promising and clinically translatable platform to augment radiotherapy induced anti‐tumor immunity, particularly for patients receiving radiotherapy in conjunction with surgical interventions.

## Graphical abstract

Radiotherapy‐treated tumor membranes are integrated with Mn‐MOF nanoadjuvants to generate Mn@RM, a lymph node‐draining nanovaccine that delivers radiotherapy‐remodeled antigenic components. Mn@RM enhances dendritic cell activation, amplifies T cell‐mediated antitumor immunity, and synergizes with radiotherapy and PD‐1 blockade for combination cancer immunotherapy.

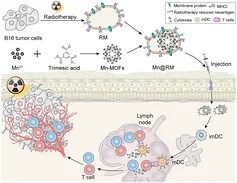

## 1 Introduction

Radiotherapy is a widely used clinical treatment modality that exerts its anti‐tumor effects primarily by inducing DNA damage in tumor cells [1]. This process not only increases the tumor mutational burden but also elevates the expression of tumor‐associated antigens, potentially generating neoantigens derived from nonsynonymous mutations [2]. Moreover, radiotherapy can trigger an “in situ vaccination effect,” in which tumor cell death and subsequent antigen release activate the adaptive immune system [3]. However, radiotherapy alone is often insufficient to induce a potent and durable anti‐tumor immune response, with limited strength and persistence observed in clinical settings [4, 5]. A major contributing factor is the immunosuppressive tumor microenvironment, which is enriched in regulatory T cells that impair the function of antigen‐presenting cells [6]. This immunosuppressive microenvironment hampers both the processing and presentation of tumor‐derived antigens and the recruitment and activation of peripheral effector T cells, thereby limiting the immunogenic potential of radiotherapy‐induced neoantigens [7].

Utilizing radiation‐derived antigens to develop personalized tumor vaccines represents a promising strategy. Personalized therapeutic cancer vaccines targeting tumor‐specific neoantigens have emerged as a promising strategy to enhance anti‐tumor immune responses [8]. To date, over 100 clinical trials investigating neoantigen‐based cancer vaccines are either ongoing or have been completed, with the majority reporting encouraging clinical outcomes [9]. For example, a clinical study in patients with high‐risk stage III/IV melanoma (NCT01970358) demonstrated that neoantigen peptide‐based vaccines could elicit both CD4^+^ and CD8^+^ T cell responses, thereby enhancing the efficacy of immune checkpoint inhibitors [10]. Another trial involving glioblastoma (NCT02149225) showed that neoantigen vaccines could induce CD4^+^ and CD8^+^ T cell responses even in immunologically “cold” tumors, leading to improved therapeutic outcomes [11]. In studies focusing on radiotherapy‐induced neoantigen vaccines, researchers from Weill Cornell Medicine and collaborators used the 4T1 murine tumor model to predict potential neoantigen peptides generated after radiotherapy. Among 293 identified somatic mutations, nine putative radiotherapy‐specific neoantigen peptides were selected for vaccine formulation. The resulting neoantigen vaccine successfully elicited both CD4^+^ and CD8^+^ T cell‐mediated anti‐tumor immune responses and significantly delayed tumor growth [12]. These studies further validated the feasibility of developing tumor vaccines based on radiotherapy‐induced neoantigens. Nevertheless, the overall process of neoantigen discovery remains in its infancy and faces significant challenges, including low efficiency and high cost [13]. Current neoantigen identification strategies mainly rely on bioinformatic analysis and MHC‐peptide binding prediction algorithms [14]. Typically, whole‐exome sequencing is used to identify genetic alterations such as single nucleotide variants, insertions, deletions, and frameshift mutations, while RNA sequencing helps prioritize candidate neoantigens [15]. The final selection of neoantigen peptide candidates is typically based on their predicted binding affinity to major histocompatibility complex (MHC) molecules. However, the development of personalized neoantigen vaccines is often time consuming requiring over three months and incurs substantial costs, frequently amounting to tens of thousands of U.S. dollars. These logistical and financial constraints are key factors contributing to the limited enrollment observed in current clinical trials of neoantigen vaccines, thereby impeding their broader clinical translation [16].

Biomimetic cancer vaccines derived from tumor cell components have attracted growing interest in the field of personalized immunotherapy [17]. This approach leverages the fact that tumor cell membranes naturally display tumor antigens that have already undergone intracellular processing and MHC/HLA presentation, thereby facilitating the induction of tumor‐specific T cell responses [18]. Such broad antigen coverage eliminates the need for precise identification and synthesis of individual neoantigens, substantially reducing the time and cost of antigen screening and validation. However, despite these advantages, membrane‐based vaccines often exhibit low immunogenicity due to the intrinsic immune evasion mechanisms of tumor cells [19]. To overcome this limitation, the rational design of adjuvants and delivery strategies is critical for achieving potent immune activation. An ideal cancer vaccine should enable the co‐delivery of antigens and adjuvants in a manner that ensures efficient lymph node drainage, promotes uptake by dendritic cells (DCs), and enhances antigen presentation, thereby initiating robust T cell responses [20]. For small‐molecule immune adjuvants, biodegradable nanoparticles such as PLGA are commonly used as carriers, onto which tumor cell membranes are coated via hydrophobic interactions to generate biomimetic nanovaccines [21]. For metal ion‐based adjuvants, including manganese, cobalt, and iron ions, metal‐organic frameworks (MOFs) have emerged as promising delivery platforms [22]. In particular, manganese‐based MOF (Mn‐MOF) nanoparticles can be effectively integrated with tumor membranes, enabling co‐delivery of antigens and adjuvants while simultaneously promoting immune activation [23].

**SCHEME 1:**
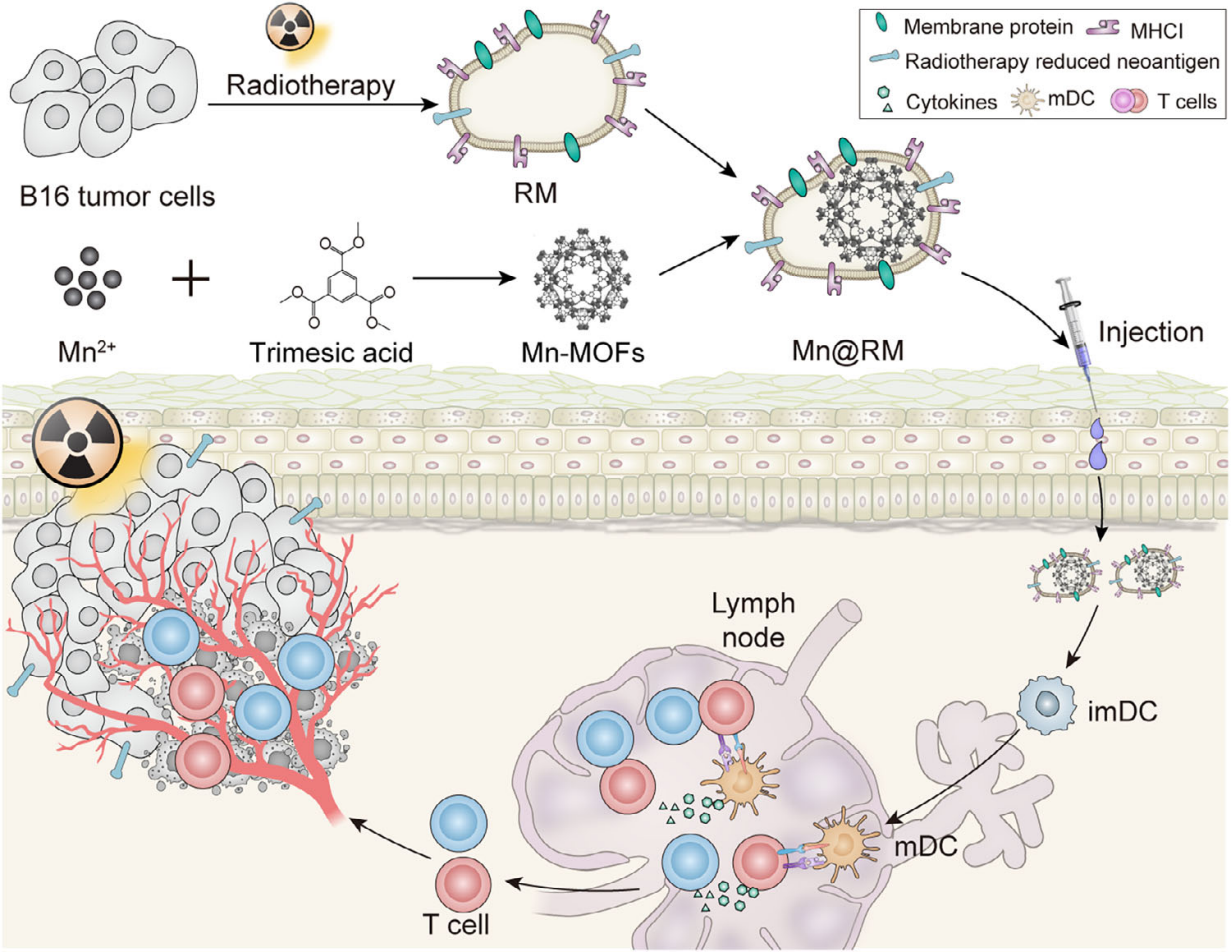
Schematic illustration of the post‐radiotherapy tumor membrane‐derived nanovaccine Mn@RM for enhancing the efficacy of cancer radiotherapy. Radiotherapy‐treated tumor membranes (RM) are integrated with Mn‐MOF nanoadjuvants to generate Mn@RM, a lymph node‐draining nanovaccine that delivers radiotherapy‐remodeled antigenic components. Mn@RM enhances dendritic cell activation, amplifies T cell‐mediated anti‐tumor immunity, and synergizes with radiotherapy and PD‐1 blockade for combination cancer immunotherapy. (MHC I: Major Histocompatibility Complex Class I, imDC: immature dendritic cells, mDC: mature dendritic cells).

In this study, we propose a therapeutic strategy that delivers radiotherapy‐induced neoantigens to lymph nodes to amplify the in situ vaccine effect of radiotherapy. To validate this approach, we first performed whole‐exome sequencing to confirm that radiation‐specific mutations induced by radiotherapy in a mouse subcutaneous tumor model can be faithfully recapitulated in a homologous tumor cell line. Building on these findings, we designed and fabricated a therapeutic cancer nanovaccine by integrating radiotherapy‐derived membranes (RM) with manganese‐based metal–organic framework (Mn‐MOF) nanoadjuvants. The resulting nanovaccine, termed Mn@RM, enables the co‐delivery of antigens and adjuvants through hydrophobic interactions between RM and Mn‐MOFs, as illustrated in Scheme 1. Owing to its nanoscale size, Mn@RM efficiently accumulates in lymph nodes following subcutaneous vaccination, ensuring that radiation‐induced neoantigens are presented in an immune cell–rich environment. Moreover, the Mn‐MOF nanoadjuvant further enhances antigen presentation by activating the cGAS–STING signaling pathway in dendritic cells (DCs). Collectively, this strategy provides a simple and clinically translatable platform with the potential to benefit patients receiving radiotherapy in combination with surgical resection.

## 2 Results and Discussion

### 2.1 Radiotherapy‐Induced Mutations can be Recapitulated Between In Vitro and In Vivo

**FIGURE 1:**
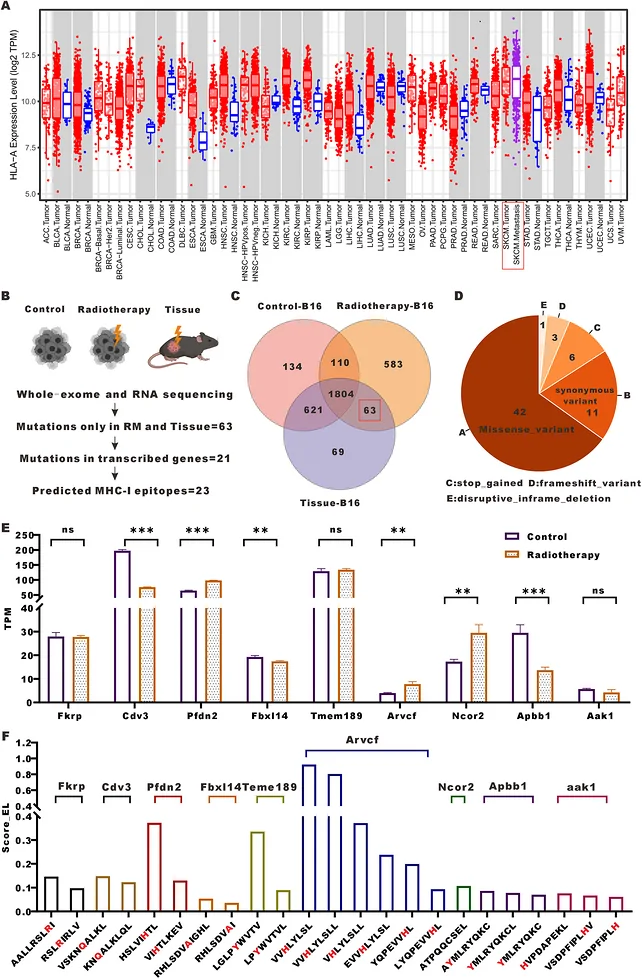
Comparative Analysis of Radiotherapy‐Induced Specific Mutations In Vitro and In Vivo. (A) Expression levels of HLA‐A across different cancer types, with skin cutaneous melanoma (SKCM) highlighted in red. (B) Schematic illustration of the workflow used to identify radiotherapy‐induced neoantigens. (n = 3) (C) Mutation profiling of wild‐type B16‐F10 cells, in vitro–irradiated B16‐F10 cells, and B16‐F10 tumor tissues following in vivo radiotherapy. (D) Classification of 63 common mutations that were exclusively detected after irradiation in both in vitro B16‐F10 cells and in vivo tumor tissues, categorized by mutation type. (E) Analysis of the expression levels of genes that can generate potential neoantigens before and after radiotherapy. (n = 3) (F) The sequence of specific neoantigen peptides induced by radiotherapy and the summary of MHC scores. All data are presented as the mean ± SEM. P‐values of the experiments were calculated by one‐way analysis of variance (ANOVA). *P < 0.05, **P < 0.01, ***P < 0.001.

For neoantigen‐based cancer vaccines, a key prerequisite for effective treatment is the expression of vaccine‐encoded neoantigen sequences at the tumor site [24]. This requirement is particularly critical for neoantigens derived from radiation‐induced mutations, given the inherent randomness of their occurrence. Therefore, the first objective of this study was to determine whether neoantigens generated by radiotherapy‐induced mutations in vitro could be recapitulated in vivo following radiotherapy. To ensure sufficient antigen presentation, tumor cell lines with high levels of membrane‐associated antigens were selected, as these are more likely to present abundant MHC–peptide complexes. Prior to conducting in vitro and in vivo validation experiments, HLA gene expression profiles across various cancer types were analyzed using data from The Cancer Genome Atlas (TCGA) to guide the selection of appropriate tumor models. As shown in Figure 1A, skin cutaneous melanoma (SKCM) exhibits high levels of HLA gene expression among 33 cancer types. Among murine melanoma models, the B16‐F10 cell line is one of the most widely used and well‐characterized syngeneic tumor models. Its compatibility with immunocompetent mice makes it ideal for evaluating tumor immunotherapy strategies. Accordingly, the B16‐F10 melanoma cell line was selected as the tumor model for subsequent investigations in this study.

To identify radiotherapy‐induced neoantigens in the B16‐F10 melanoma model, whole‐exome sequencing (WES) and RNA sequencing (RNA‐seq) were performed on three groups of samples: untreated wild‐type B16‐F10 cells (Control‐B16), B16‐F10 cells exposed to radiation in vitro (Radiotherapy‐B16), and subcutaneous B16‐F10 tumors collected from irradiated mice (Figure 1B). Comparative analysis of DNA mutations among these groups revealed that in vitro irradiation of B16‐F10 cells generated 646 unique somatic mutations, while irradiation of tumor‐bearing mice induced 132 new mutations in tumor tissues. Among the 646 radiation‐associated novel mutations identified in vitro and the 132 detected in vivo, 63 overlapping mutations were identified between the two datasets. Given that mutations arising from differences in growth conditions would be expected to differ between the in vivo and in vitro systems, these shared mutations were unlikely to reflect environment‐dependent alterations. Instead, their consistent detection across three independent experimental groups supports their classification as reproducible, radiation‐induced, tumor‐specific mutations. These findings provide evidence that radiation‐induced specific mutational events can be reproducibly detected in both in vivo tumor models and in vitro cultured tumor cell models (Figure 1C). Among these 63 overlapping mutations, 42 were missense variants, 11 were synonymous variants, 6 were stop‐gained mutations, 3 were frameshift variants, and 1 was a disruptive in‐frame deletion (Figure 1D). Excluding the synonymous variants, the remaining categories represent mutation types with the potential to generate neoantigenic peptides. To evaluate their immunogenic potential, RNA expression levels of the 52 nonsynonymous mutated genes were analyzed. RNA‐seq data showed that 21 of these genes were transcriptionally active in both B16‐F10 cells and subcutaneous tumor tissues. To further investigate their potential to generate MHC class I–restricted neoantigens, the NetMHC algorithm was used to predict MHC‐I binding affinities of 8–11‐mer peptide sequences encompassing the mutation sites. Among the 21 expressed mutations, 9 genes (Fkrp, Cdv3, Pfdn2, Fbxl14, Tmem189, Arvcf, Ncor2, Apbb1, and Aak1) were predicted to encode peptides with high binding affinity to MHC‐I molecules, suggesting their potential as radiotherapy‐induced neoantigens. Expression dynamics of these genes in response to radiotherapy were further examined: Fkrp, Tmem189, and Aak1 expression remained unchanged; Pfdn2, Arvcf, and Ncor2 were upregulated; while Cdv3, Fbxl14, and Apbb1 were down regulated following irradiation (Figure 1E). To identify candidate neoepitopes, a 2% rank threshold for weak binders was applied according to NetMHCpan 4.1 guidelines. All predicted MHC‐I–binding peptide sequences are listed in Figure 1F, with mutation sites highlighted in red. To further evaluate whether this radiotherapy‐induced mutation and candidate neoantigen prediction strategy was applicable beyond the B16F10 melanoma model, a similar WES/RNA‐seq‐based analysis was performed in the Lewis lung carcinoma model. Untreated Lewis cells, in vitro irradiated Lewis cells, and irradiated Lewis tumor tissues were subjected to comparative mutation analysis and transcriptomic filtering (Figure S1A). Consistent with the B16F10 results, irradiated Lewis cells and irradiated Lewis tumor tissues shared a subset of radiation‐associated mutations, with 273 overlapping mutations identified between the two irradiated conditions (Figure S1B). Among these shared mutations, 173 were missense variants, 97 were synonymous variants, and 3 were stop‐gained mutations (Figure S1C). After excluding synonymous variants and integrating RNA expression analysis, 25 transcribed mutant genes were retained for candidate neoantigen evaluation. Several expressed genes, including Hjurp and Herpud1, were detected in both irradiated Lewis cells and irradiated tumor tissues (Figure S1D). NetMHCpan‐based prediction further identified 12 candidate MHC‐I binding epitopes derived from expressed shared mutant genes (Figure S1E). Collectively, these results demonstrate that radiotherapy can induce reproducible, specific mutations capable of generating neoantigenic peptides, and that such mutations can be detected in both in vitro cell models and in vivo tumor tissues. These findings provide theoretical support for the development of radiotherapy‐matched tumor membrane vaccines, highlighting their potential to synergize with radiotherapy in enhancing anti‐tumor immunity.

### 2.2 Fabrication of the Mn@RM Nanovaccine by Integrating Post‐Radiotherapy Tumor Cell Membranes With Mn‐MOF Nanoadjuvants

Adjuvants play a critical role in enhancing the immunogenicity of cancer vaccines [25]. Among them, STING agonists, which trigger type I interferon (IFN‐γ) responses, have been shown to significantly promote antigen‐specific CD8^+^T cell activation and are considered ideal partners for neoantigen‐based vaccines [26]. In particular, manganese ions (Mn^2^^+^) have been identified as potent activators of the cGAS–STING signaling pathway [27]. Metal‐organic frameworks (MOFs), a class of nanomaterials composed of metal ions and organic ligands, possess hydrophobic surfaces that facilitate interactions with lipid bilayers [28]. This unique property enables efficient integration of tumor cell membranes onto Mn‐MOFs surfaces, thereby allowing co‐delivery of tumor‐associated antigens and Mn‐based immunostimulatory agents.

**FIGURE 2:**
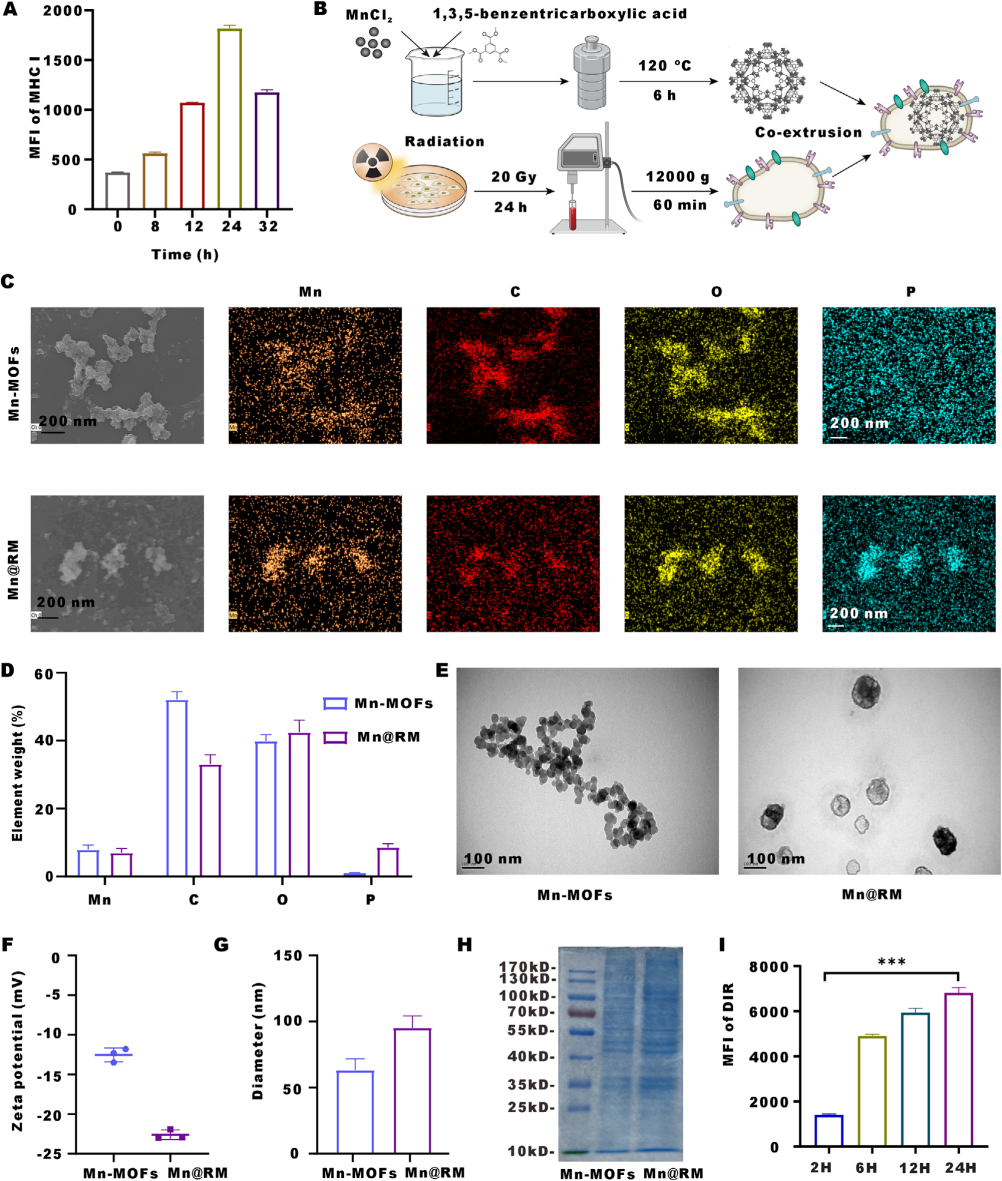
Preparation and Characterization of Mn@RM Nanovaccine. (A) Expression levels of MHC class I molecules on B16‐F10 cells at various time points following radiotherapy. (n = 3) (B) Schematic illustration of the fabrication process for the Mn@RM nanovaccine, integrating Mn‐MOFs with irradiated tumor cell membranes. (C) Scanning electron microscopy (SEM) and Energy‐dispersive X‐ray spectroscopy (EDS) elemental mapping images of Mn‐MOFs and Mn@RM nanoparticles. Scale bar: 200 nm. (D) Elemental content ratios of Mn, C, O, and P in Mn‐MOFs and Mn@RM. (n = 3) (E) Transmission electron microscopy (TEM) images showing the morphology and membrane coating of Mn‐MOFs and Mn@RM. Scale bar: 200 nm. (F) The zeta potential of Mn‐MOFs and Mn@RM. (n = 3) (G) The hydrodynamic size of Mn‐MOFs and Mn@RM. (n = 3) (H) SDS–PAGE analysis comparing protein profiles of Mn‐MOFs and Mn@RM. (I) Flow cytometric analysis of Mn@RM uptake by BMDCs (n = 3). All data are presented as the mean ± SEM. P‐values of the experiments were calculated by one‐way analysis of variance (ANOVA). *P < 0.05, **P < 0.01, ***P < 0.001.

A key parameter in isolating RM is the timing of membrane extraction following radiotherapy, as antigen presentation is largely dependent on the expression levels of MHC class I molecules. To determine the optimal harvesting time, the surface expression of MHC‐I on B16‐F10 cells at various time points post‐irradiation were analyzed. The data showed that MHC‐I expression peaked at 24 h post‐radiotherapy, which was subsequently chosen as the optimal time point for RM extraction (Figure 2A). The synthesis of the Mn@RM nanovaccine is illustrated in Figure 2B. Briefly, Mn‐MOF nanoparticles were synthesized via hydrothermal method, and RMs were prepared using ultrasonic lysis. The final Mn@RM nanovaccine was fabricated via a co‐extrusion process. Scanning electron microscopy (SEM) images (Figure 2C) revealed that Mn@RM nanoparticles exhibited a rougher surface morphology compared with Mn‐MOFs, indicating successful membrane coating. Elemental mapping via energy‐dispersive X‐ray spectroscopy was employed to verify membrane coating. Mn‐MOFs were composed of Mn, C, and O elements, whereas Mn@RM particles additionally contained phosphorus (P), a hallmark of phospholipid membranes. Quantitative elemental analysis (Figure 2D) further confirmed the presence of P exclusively in Mn@RM, reinforcing the successful incorporation of RM onto the nanoparticle surface. Transmission electron microscopy (TEM) images (Figure 2E) showed that Mn‐MOFs were spherical with diameters less than 100 nm. In Mn@RM group, membrane encapsulation around Mn‐MOF cores was clearly observed, with multiple particles visibly embedded within lipid membranes, confirming successful fabrication. Zeta potential measurements (Figure 2F) showed a shift from −12.5 ± 0.87 mV for Mn‐MOFs to −22.6 ± 0.60 mV for Mn@RM, consistent with surface coating by negatively charged cell membranes. Dynamic light scattering (DLS) analysis (Figure 2G) revealed that Mn@RM had a slightly larger hydrodynamic diameter than uncoated Mn‐MOFs, further supporting successful membrane integration. To assess whether the membrane coating retained its native protein content, SDS–PAGE analysis was performed to compare the protein profiles of RM and Mn@RM (Figure 2H). The results demonstrated that Mn@RM retained the characteristic protein bands of RM, suggesting preservation of membrane‐associated proteins. Finally, the cellular uptake of Mn@RM by CD11C^+^ dendritic cells was evaluated using flow cytometry. In Figure 2I, the results indicated time‐dependent uptake, confirming efficient internalization of Mn@RM by dendritic cells. Together, these physicochemical and biological characterizations demonstrate that RM was successfully incorporated onto Mn‐MOF nanoparticles to generate a structurally stable and immunologically functional Mn@RM nanovaccine.

### 2.3 Mn@RM Activates the cGAS‐STING Signaling Pathway in DCs

**FIGURE 3:**
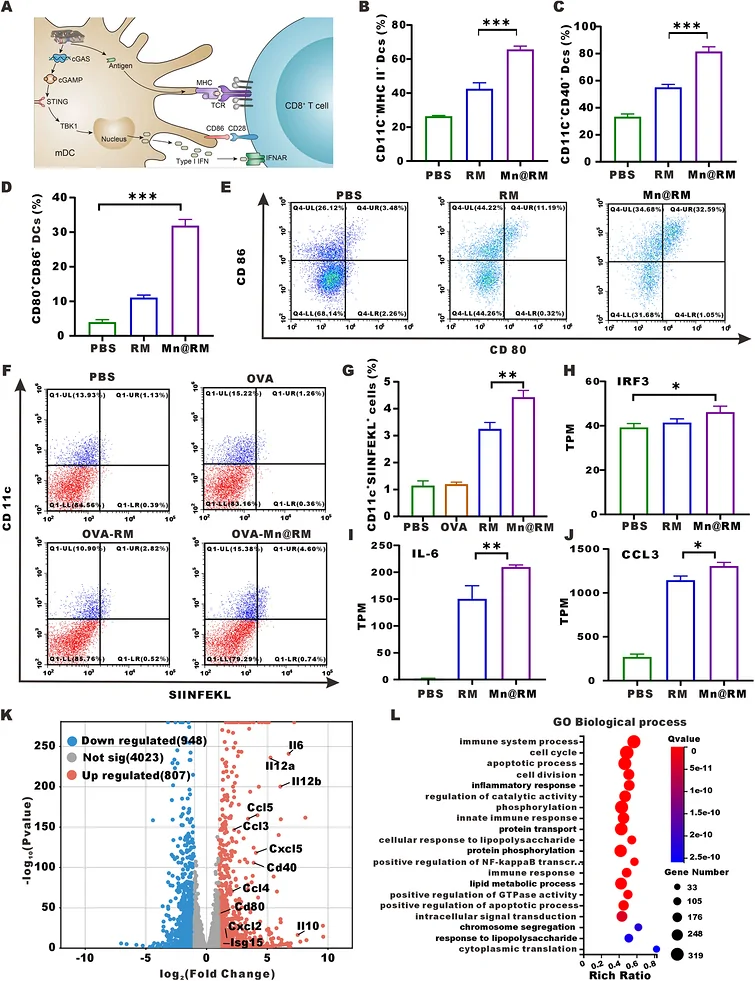
In vitro validation of cGAS‐STING pathway activation by Mn@RM in BMDCs. (A) Schematic diagram illustrating the activation of the cGAS‐STING signaling pathway in BMDCs. (B‐D) Flow cytometry analysis of surface expression levels of antigen‐presenting and co‐stimulatory molecules on BMDCs following treatment with PBS, RM, or Mn@RM: MHC class II (B), CD40 (C), and CD80/CD86 (D). (n = 3) (E) Representative flow cytometry gating plots of BMDCs from the PBS, RM, and Mn@RM groups. (F) Representative dot plots showing SIINFEKL peptide presentation by BMDCs after co‐culture with OVA‐expressing B16 cells in the PBS, RM, and Mn@RM groups.(G) Quantitative analysis of antigen presentation efficiency based on SIINFEKL expression levels. (H‐J) The effects of RM and Mn@RM on the expression levels of IRF3, IL‐6 and CCL3 in BMDC cells. (n = 3) (K) Volcano plot showing differentially expressed genes in BMDCs following co‐culture with Mn@RM. (L) Gene ontology (GO) enrichment analysis of upregulated genes involved in immune‐related signaling pathways after Mn@RM treatment. All data are presented as the mean ± SEM. P‐values of the experiments were calculated by one‐way analysis of variance (ANOVA). *P < 0.05, **P < 0.01, ***P < 0.001.

The cGAS‐STING pathway is a critical component of the innate immune system, as it enhances antigen presentation and promotes the production of type I interferons and pro‐inflammatory cytokines, thereby facilitating the activation and recruitment of other immune cells [29]. Activation of the cGAS‐STING signaling in DCs can robust secretion of type I interferons, which in turn enhances the anti‐tumor immune response elicited by tumor vaccines (Figure 3A). This part of the study aimed to determine whether the combination of Mn‐MOFs nanoadjuvants with RM vaccine could enhance the antigen‐presenting efficiency of DCs via activation of the cGAS‐STING pathway. First, the effects of RM vaccine alone and in combination with Mn‐MOFs nanoadjuvants on the surface expression of MHC II, CD40, CD80, and CD86 on DCs were tested. Among these markers, the expression level of MHC II directly reflects the antigen loading capacity and antigen‐presenting efficiency of DCs. CD40, CD80, and CD86 are co‐stimulatory molecules expressed on antigen‐presenting cells (APCs) that interact with T cells. Specifically, CD40 binds to CD40L on T cells, while CD80 and CD86 bind to CD28 on T cells—interactions that are critical for effective T cell activation. As shown in Figure 3B, MHC II expression was increased from 26.38 ± 0.45% in the PBS group to 42.54 ± 3.48% in the RM group, and was further enhanced to 65.70 ± 1.87% in the Mn@RM group. This result indicates that Mn@RM markedly improved the antigen‐presenting capacity of BMDCs compared with RM alone (p = 0.0009). Consistently, CD40 expression showed a similar trend. As shown in Figure 3C, the proportion of CD40^+^ BMDCs was 33.37 ± 1.08% in the PBS group, 55.07 ± 1.24% in the RM group, and 81.57 ± 1.99% in the Mn@RM group. In addition, the proportion of CD80^+^CD86^+^ BMDCs increased from 3.98 ± 0.41% in the PBS group to 11.06 ± 0.42% in the RM group, and was further elevated to 31.89 ± 1.03% after Mn@RM treatment (Figure 3D). These results demonstrate that the Mn@RM nanovaccine, incorporating Mn‐based nanoadjuvants, substantially enhances the antigen presentation capacity of DCs. Representative flow cytometry gating plots for PBS, RM, and Mn@RM groups are shown in Figure 3E.

To further investigate the effect of Mn‐based nanoadjuvants on enhancing the antigen presenting capacity of DCs, cell membranes were extracted from these B16‐OVA cells to construct a Mn@RM‐OVA nanovaccine, which was then co‐cultured with DCs. The level of SIINFEKL epitope peptide presented on the surface of DCs was assessed. Figure 3F shows representative flow cytometry gating plots for DCs incubated with PBS, free OVA protein, OVA‐RM, and OVA‐Mn@RM. Quantitative analysis of SIINFEKL in DCs expression was presented in Figure 3G, free OVA treatment did not markedly increase SIINFEKL‐H‐2^Kb^ presentation compared with PBS, with SIINFEKL positive BMDCs remaining at 1.20 ± 0.07% in the OVA group and 1.14 ± 0.17% in the PBS group. In contrast, OVA‐RM markedly increased SIINFEKL‐H‐2^Kb^ presentation to 3.25 ± 0.24%, while OVA‐Mn@RM further enhanced this level to 4.43 ± 0.25%, which was significantly higher than that in the OVA‐RM group (p = 0.0023). These results demonstrate that RM facilitates antigen presentation by BMDCs and that Mn‐MOF nanoadjuvants further improve RM‐mediated antigen processing and presentation, and the partial antigen presentation observed in the RM and Mn@RM group may be associated with membrane‐associated antigens together with endogenous danger‐associated signals retained or exposed in radiotherapy‐treated tumor cell membranes. More importantly, the further enhancement observed in the Mn@RM group indicates that Mn‐MOF nanoadjuvants can promote RM‐mediated antigen presentation and DC activation.

To investigate whether this enhancement was associated with immune signaling activation, RNA‐seq analysis was performed in BMDCs treated with PBS, RM, or Mn@RM. As shown in Figure 3H, IRF3 expression increased from 39.22 ± 1.03 in the PBS group to 41.43 ± 0.97 in the RM group and 46.16 ± 1.56 in the Mn@RM group (p = 0.0161), suggesting activation of cGAS‐STING associated transcriptional signaling [30]. Consistently, IL‐6, an inflammatory cytokine upregulated via NF‐κB signaling following cGAS‐STING activation, plays essential roles in DC maturation, T cell activation, and propagation of the inflammatory response, was strongly upregulated from 1.91 ± 0.43 in the PBS group to 150.00 ± 14.00 in the RM group and 209.67 ± 2.19 in the Mn@RM group (Figure 3I). CCL3 is a chemokine secreted by activated BMDCs, primarily responsible for recruiting T cells and other immune cells to sites of inflammation, also increased from 271.00 ± 18.25 in the PBS group to 1162.00 ± 26.08 in the RM group and 1306.33 ± 24.10 in the Mn@RM group (Figure 3J). Together, these findings demonstrate that Mn@RM not only enhances RM‐mediated antigen presentation and DC maturation, but also promotes immune activation‐associated gene expression in BMDCs, supporting its function as an effective tumor membrane‐based nanovaccine platform.

To provide a more comprehensive view of the immunological impact of Mn@RM internalization by DCs, the key genes upregulated by Mn@RM and their associated signaling pathways were analyzed by RNA‐seq. The volcano plot in Figure 3K highlights several significantly upregulated inflammatory and chemotactic factors, including IL‐6, IL‐12A, IL‐12B, IL‐10, CCL3, CCL4, CCL5, CXCL12, and CXCL15, all of which play vital roles in immune responses and inflammation regulation. Their upregulation indicates strong immune activation and the establishment of an inflammatory microenvironment. Gene Ontology (GO) biological process analysis further revealed that the upregulated genes were significantly enriched in immune‐related pathways, including immune system process, inflammatory response, and innate immune response (Figure 3L). Collectively, these findings demonstrate not only the enhanced immunostimulatory effects of Mn‐MOFs nanoadjuvants when combined with RM, but also the strong immunogenicity of Mn@RM. These results provide solid theoretical evidence supporting Mn@RM as a promising therapeutic cancer vaccine.

### 2.4 Mn@RM Exhibits Excellent Biosafety and Immunogenicity In Vivo

**FIGURE 4:**
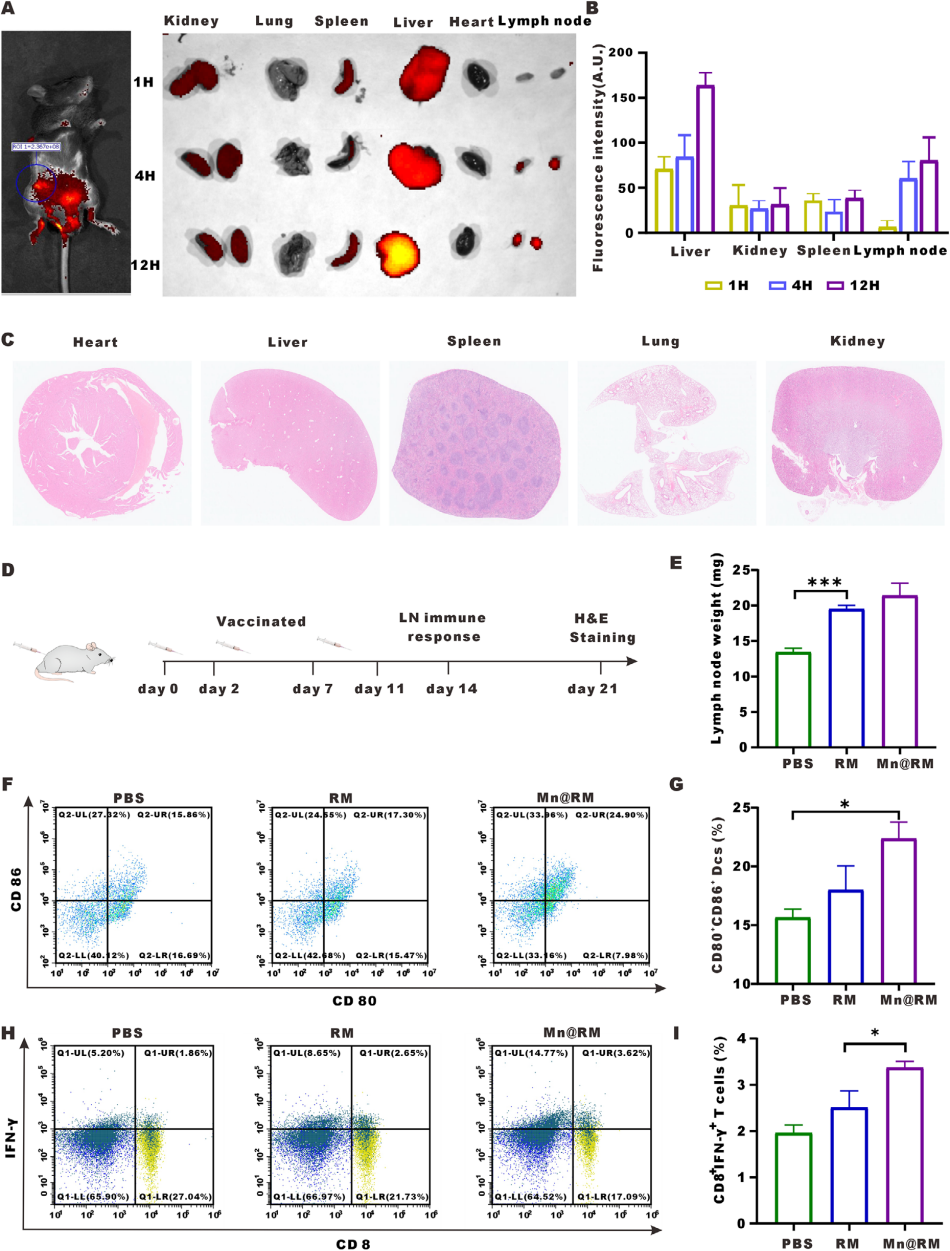
Biodistribution, biosafety, and modulation of the lymph node immune microenvironment by Mn@RM. (A) In vivo fluorescence imaging of healthy mice subcutaneously injected with DiR‐labeled Mn@RM at various time points post‐vaccination (1, 4, and 12 h). (B) Quantitative analysis of fluorescence intensity in major organs, including the liver, kidneys, spleen, and lymph nodes. (n = 3) (C) Representative H&E staining of heart, liver, spleen, lung, and kidney tissues collected three weeks post‐vaccination to evaluate potential pathological changes. (D) Schematic illustration of the overall biosafety and immunogenic properties of Mn@RM nanovaccine (n = 3). (E) Quantitative analysis of lymph node weight in mice vaccinated with PBS, RM, or Mn@RM. (n = 3) (F–G) Flow cytometry analysis of the expression levels of co‐stimulatory molecules CD80 and CD86 on DCs isolated from inguinal lymph nodes after vaccination. (n = 3) (H–I) Flow cytometric analysis of the proportion of activated CD8^+^ IFN‐γ^+^ T cells in lymph nodes following vaccination with PBS, RM, or Mn@RM. (n = 3) All data are presented as mean ± SEM. Statistical analysis was performed using one‐way ANOVA. *p < 0.05, **p < 0.01, ***p < 0.001.

Lymph nodes serve as central hubs for anti‐tumor immune responses triggered by therapeutic cancer vaccines [31]. They are critical sites where DCs present antigens and activate T cells. The immunological microenvironment and functional status of lymph nodes directly influence the extent of vaccine‐induced anti‐tumor immunity [32]. This work focuses on delivering radiotherapy derived neoantigen peptides to lymph nodes to stimulate a robust anti‐tumor immune response. To assess the in vivo distribution of Mn@RM, the biodistribution of DiR‐labeled Mn@RM was analyzed at 1, 4, and 12 h after subcutaneous injection. Figure 4A revealed that Mn@RM primarily accumulated in the kidneys, liver, spleen, and lymph nodes within 12 h post‐vaccination, with noticeable accumulation in lymph nodes observed as early as 4 h. Figure 4B provides quantitative analysis of Mn@RM distribution across major organs over time. This distribution pattern is likely attributable to the abundance of lymphatic vessels in subcutaneous tissues, which facilitate vaccine transport to the lymphatic system and subsequently to the lymph nodes. The presence of Mn@RM in the liver and kidneys may be explained by the return of lymphatic fluid to systemic circulation. To evaluate potential off‐target toxicity, histological analysis of major organs (heart, liver, spleen, lung, and kidney) was performed three weeks after Mn@RM vaccination. As shown in Figure 4C, hematoxylin and eosin (H&E) staining revealed no abnormal morphology in any examined tissues, indicating that Mn@RM does not induce pathological damage and is well tolerated in vivo. The schematic timeline of vaccination and H&E staining protocol is shown in Figure 4D. Briefly, C57BL/6 mice were subcutaneously immunized with Mn@RM on days 0, 2, 7, and 11. On day 14, inguinal lymph nodes adjacent to the injection site were harvested for analysis of the local immune microenvironment, and the H&E staining was analyzed at day 21.

Figure 4E shows the weights of lymph nodes from mice vaccinated with PBS, RM, or Mn@RM. As key immune organs, lymph node enlargement often reflects immune cell expansion and local inflammation, serving as an indicator of immune activation. The data indicate that both RM and Mn@RM significantly increased lymph node mass, suggesting strong immunogenicity of RM‐based therapeutic cancer vaccines. Figure 4F illustrates the maturation levels of DCs in lymph nodes following vaccination. DC maturation was significantly upregulated in both RM and Mn@RM groups compared with PBS. As shown in Figure 4G, the proportion of mature DCs was 15.65 ± 0.72% in the PBS group, 17.34 ± 1.43% in the RM group, and 22.38 ± 1.41% in the Mn@RM group. Although RM alone showed a mild increase in DC maturation compared with PBS, Mn@RM induced a significantly higher level of DC maturation than PBS (p = 0.0198), consistent with the enhanced antigen presenting effect observed in vitro. The activation of CD8^+^ T cells in lymph nodes was further assessed by measuring the proportion of CD8^+^IFN‐γ^+^ T cells. As shown in Figure 4H,I, the proportion of CD8^+^IFN‐γ^+^ T cells was 1.96 ± 0.17% in the PBS group, 2.51 ± 0.36% in the RM group, and 3.38 ± 0.13% in the Mn@RM group. Both RM and Mn@RM increased CD8^+^IFN‐γ^+^ T cell responses compared with PBS, while Mn@RM induced a significantly higher proportion of CD8^+^IFN‐γ^+^ T cells than RM alone (p = 0.0436). These results indicate that Mn@RM effectively promotes lymph node immune activation by enhancing DC maturation and cytotoxic T cell activation, supporting its potential as an immunogenic therapeutic nanovaccine.

To further evaluate whether vaccination with RM or Mn@RM could induce T cells responsive to radiotherapy‐treated tumor membrane‐associated antigens, an ex vivo RM re‐stimulation assay was performed. Mice were vaccinated with PBS, CM, RM, or Mn@RM, and splenic T cells were isolated 14 days after vaccination. The isolated cells were then co‐cultured with RM as the antigenic stimulus, and CD8^+^T cell activation was assessed by measuring CD69 expression using flow cytometry. As shown in Figure S2, RM re‐stimulation induced different levels of CD8^+^CD69^+^T cell activation among the four vaccination groups. The proportion of CD8^+^CD69^+^T cells was 3.44±0.11% in the PBS group, 4.19±0.33% in the CM group, 4.25±0.37% in the RM group, and 5.05±0.26% in the Mn@RM group. Compared with the PBS group, RM vaccination showed an increasing trend in CD8^+^CD69^+^T cell activation, although the difference did not reach statistical significance (p = 0.0734). In contrast, Mn@RM vaccination induced a significantly higher proportion of CD8^+^CD69^+^T cells than PBS (p = 0.0005). These results suggest that RM carried antigenic components may contribute to T cell activation (Figure S2), while the incorporation of Mn‐MOF nanoadjuvants significantly enhances this immune response. Although this assay does not define the contribution of each individual predicted neoantigen peptide, it provides functional evidence that the complete antigenic repertoire of RM can participate in T cell activation. These findings further support the potential of radiotherapy‐treated tumor cell membranes as an immunogenic antigen source for combination with radiotherapy.

### 2.5 Mn@RM Enhances the Therapeutic Efficacy of Radiotherapy In Vivo

**FIGURE 5:**
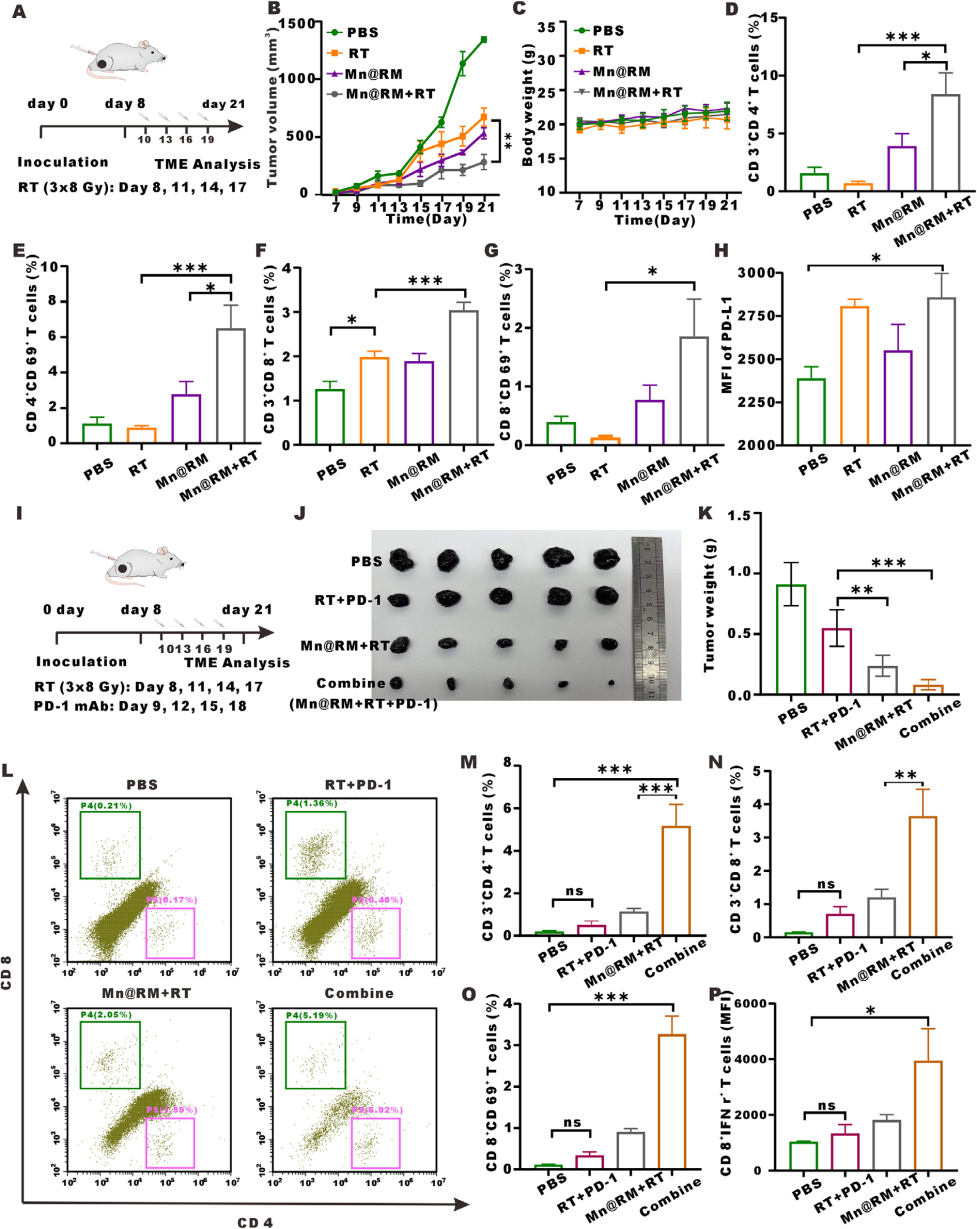
Study on the anti‐tumor synergistic effect of radiotherapy and PD‐1 monoclonal antibody and Mn@RM. (A) Schematic of subcutaneously implanted B16F10 tumor treatment with PBS, RT (3×8 Gy), Mn@RM and Mn@RM + RT. (n = 5) (B) Tumor volume of different groups during the treatment. (C) The change of body weight during the treatment. (D, E) Infiltration ratio and activation level of CD4^+^ T cells in the tumor microenvironment after treatment. (F, G) Infiltration ratio and activation level of CD8^+^ T cells in the tumor microenvironment after treatment. (H) Expression level of PD‐L1 on the surface of tumor cells in the tumor microenvironment after treatment. (I) Schematic of subcutaneously implanted B16F10 tumor treatment by PBS, RT+PD‐1 mAb, Mn@RM + RT and Mn@RM + PD‐1 mAb + RT (Combine group). (n = 5). (J) The image of tumors in the above four groups after treatment. (K) The tumor mass of the above four groups at the end of treatment. (L‐P) Immune analysis of the tumor microenvironment in the above four groups after treatment, (M) Proportion of infiltrating CD4^+^ T cells, (N) Proportion of infiltrating CD8^+^ T cells, (O) Proportion of infiltrating CD8^+^CD69^+^ T cells, (P) Proportion of infiltrating CD8^+^IFN γ^+^ T cells. All data are presented as the mean ± SEM. P‐values of the experiments were calculated by one‐way analysis of variance (ANOVA). *P < 0.05, **P < 0.01, ***P < 0.001.

To evaluate whether the Mn@RM nanovaccine can enhance the anti‐tumor efficacy of radiotherapy, this in vivo study first compared the therapeutic effects of radiotherapy, Mn@RM, and Mn@RM combined with radiotherapy in the B16 subcutaneous tumor model. The experimental design is outlined in Figure 5A. Briefly, C57BL/6 mice were subcutaneously inoculated with 2 × 10^5^ B16‐F10 melanoma cells. Starting on day 8 post inoculation, mice received radiotherapy on days 8, 11, 14, and 17, and were vaccinated with Mn@RM on days 10, 13, 16, and 19. After four treatment cycles, the synergistic anti‐tumor effect of Mn@RM and RT was evaluated. As shown in Figure 5B, Mn@RM combined with radiotherapy produced the most pronounced tumor growth suppression among all treatment groups. On day 21, the average tumor volume reached 1343.10 ± 23.54 mm^3^ in the PBS group, whereas RT alone reduced tumor volume to 672.30 ± 78.57 mm^3^, corresponding to a 49.94% inhibition of tumor growth compared with PBS. Mn@RM treatment alone further reduced tumor volume to 533.00 ± 47.39 mm^3^, corresponding to a 60.32% inhibition compared with PBS. Notably, the Mn@RM + RT group showed the strongest anti‐tumor effect, with an average tumor volume of 282.74 ± 65.33 mm^3^, corresponding to a 78.95% inhibition of tumor growth compared with PBS. In addition, Mn@RM + RT reduced tumor volume by 57.94% compared with RT alone, with a statistically significant difference between these two groups (p = 0.014). These results demonstrate that Mn@RM markedly enhances the anti‐tumor efficacy of radiotherapy, supporting its potential as a therapeutic nanovaccine for combination with RT. Figure 5C shows the body weight changes of mice across all treatment groups during the therapeutic regimen. The stable body weights suggest that Mn@RM administration does not induce overt systemic toxicity and is well tolerated in vivo.

To explore the immunological mechanisms underlying the enhanced anti‐tumor efficacy of Mn@RM combined with radiotherapy, the tumor immune microenvironment was analyzed by flow cytometry. As shown in Figure 5D, the proportion of CD4^+^T cells in the tumor microenvironment was 1.58 ± 0.51% in the PBS group, 0.71 ± 0.16% in the RT group, 3.92 ± 1.06% in the Mn@RM group, and 8.41 ± 1.82% in the Mn@RM + RT group. These results indicate that Mn@RM, particularly when combined with RT, increased CD4^+^T cell infiltration in the tumor microenvironment. The activation status of CD4^+^T cells was further evaluated by CD69 expression. As shown in Figure 5E, the proportion of CD4^+^CD69^+^T cells was 1.12 ± 0.35% in the PBS group, 0.90 ± 0.09% in the RT group, 2.78 ± 0.71% in the Mn@RM group, and 6.54 ± 1.31% in the Mn@RM + RT group. These data suggest that Mn@RM + RT promoted not only CD4^+^T cell infiltration but also CD4^+^T cell activation within the tumor microenvironment. CD8^+^T cell infiltration was also enhanced after combination treatment. As shown in Figure 5F, the proportion of CD8^+^T cells was 1.26 ± 0.17% in the PBS group, 1.98 ± 0.13% in the RT group, 1.95 ± 0.16% in the Mn@RM group, and 3.09 ± 0.18% in the Mn@RM + RT group. The Mn@RM + RT group showed the highest CD8^+^T cell infiltration, with a statistically significant increase compared with the RT group (p = 0.009). In addition, CD8^+^T cell activation was assessed by the proportion of CD8^+^CD69^+^T cells. As shown in Figure 5G, CD8^+^CD69^+^T cells accounted for 0.39 ± 0.10% in the PBS group, 0.13 ± 0.03% in the RT group, 0.75 ± 0.25% in the Mn@RM group, and 1.90 ± 0.64% in the Mn@RM + RT group. Importantly, Mn@RM + RT significantly increased activated CD8^+^T cells compared with RT alone (p = 0.0132), supporting the immunostimulatory effect of Mn@RM in the context of radiotherapy. To further evaluate the interaction between radiotherapy induced immune activation and immune suppression, PD‐L1 expression on tumor cells was analyzed. As shown in Figure 5H, PD‐L1 expression was 2389.00 ± 66.76 in the PBS group, 2798.40 ± 47.70 in the RT group, 2551.50 ± 150.99 in the Mn@RM group, and 2859.50 ± 137.47 in the Mn@RM + RT group. Both RT and Mn@RM + RT increased PD‐L1 expression in tumor cells, suggesting that radiotherapy associated immune activation may be accompanied by PD‐L1 mediated immunosuppressive feedback. Together, these results indicate that Mn@RM synergizes with radiotherapy to enhance CD4+and CD8^+^T cell infiltration and activation in the tumor microenvironment, while also providing a rationale for further combination with PD‐1/PD‐L1 immune checkpoint blockade.

### 2.6 PD‐1 Blockade Further Enhances the Anti‐Tumor Efficacy of Mn@RM Combined With RT

Radiotherapy has been shown to closely interact with the PD‐L1 expression on tumor cells [33]. Given this background, whether the combination of a PD‐1 immune checkpoint inhibitor could augment the anti‐tumor efficacy of Mn@RM plus RT was investigated. The treatment timeline was illustrated in Figure 5I. Mn@RM and RT were administered following the same schedule described previously, while anti–PD‐1 antibody was intraperitoneally injected on days 9, 12, 15, and 18. Representative tumor images from the PBS, RT + PD‐1, Mn@RM + RT, and Mn@RM + RT + PD‐1 groups are shown in Figure 5J. Tumor regression was most prominent in the triple combination group (Mn@RM + RT + PD‐1), indicating a synergistic therapeutic effect. Quantitative analysis of tumor weights post‐treatment is shown in Figure 5K. The data confirm that the addition of PD‐1 blockade significantly enhanced the therapeutic efficacy of Mn@RM + RT (p = 0.0001), further validating the benefit of immune checkpoint inhibition.

To further elucidate the immunological mechanisms underlying the enhanced anti‐tumor efficacy of the triple combination therapy, tumor infiltrating lymphocytes were analyzed by flow cytometry. As shown in Figure 5M, the proportion of CD4^+^T cells in the tumor microenvironment was 0.21 ± 0.01% in the PBS group, 0.51 ± 0.19% in the RT + PD‐1 group, 1.14 ± 0.14% in the Mn@RM + RT group, and 5.17 ± 1.01% in the Mn@RM + RT + PD‐1 group. These data indicate that Mn@RM combined with RT increased CD4^+^T cell infiltration, and this effect was further enhanced by PD‐1 blockade. A similar trend was observed for CD8^+^T cell infiltration. As shown in Figure 5N, the proportion of CD8^+^T cells was 0.15 ± 0.01% in the PBS group, 0.70 ± 0.22% in the RT + PD‐1 group, 1.21 ± 0.24% in the Mn@RM + RT group, and 3.66 ± 0.81% in the Mn@RM + RT + PD‐1 group. These results suggest that the addition of PD‐1 blockade further promoted cytotoxic T cell infiltration in tumors treated with Mn@RM and RT. The activation status of intratumoral CD8^+^T cells was further assessed by CD69 expression. As shown in Figure 5O, the proportion of CD8^+^CD69^+^T cells was 0.11 ± 0.01% in the PBS group, 0.35 ± 0.09% in the RT + PD‐1 group, 0.91 ± 0.08% in the Mn@RM + RT group, and 3.27 ± 0.44% in the Mn@RM + RT + PD‐1 group. The triple combination group showed a significantly higher proportion of activated CD8^+^CD69^+^T cells than the other treatment groups (p < 0.0001), indicating that PD‐1 blockade markedly strengthened the CD8^+^T cell activation induced by Mn@RM combined with RT. Finally, the effector function of CD8^+^T cells was evaluated by IFN‐γproduction. As shown in Figure 5P, IFN‐γexpression in intratumoral CD8^+^T cells was 1041.60 ± 10.81 in the PBS group, 1339.20 ± 307.70 in the RT + PD‐1 group, 1817.60 ± 193.65 in the Mn@RM + RT group, and 3947.20 ± 1149.08 in the Mn@RM + RT + PD‐1 group. The triple combination significantly increased IFN‐γ production compared with Mn@RM + RT (p = 0.0170), suggesting enhanced cytotoxic effector function of CD8^+^T cells. Collectively, these results demonstrate that PD‐1 blockade further potentiates the anti‐tumor immune response induced by Mn@RM combined with radiotherapy. This enhancement is associated with increased infiltration of CD4^+^ and CD8^+^T cells, elevated CD8^+^T cell activation, and enhanced IFN‐γproduction in the tumor microenvironment. These findings support the rationale that PD‐1 blockade can relieve radiotherapy‐associated immunosuppressive feedback and further amplify Mn@RM‐mediated anti‐tumor cellular immunity. In the present study, Mn@RM vaccination was administered after tumor establishment and after the initiation of radiotherapy to evaluate its therapeutic potential in an established B16‐F10 tumor model. This schedule was selected because Mn@RM is designed as a therapeutic tumor vaccine using radiotherapy‐treated tumor cell membranes as the antigen source. Vaccination before tumor implantation or at a very early stage after tumor inoculation may introduce prophylactic or non‐specific immune effects and interfere with tumor engraftment, making it difficult to assess the therapeutic efficacy of Mn@RM against established tumors. Nevertheless, the timing of vaccination relative to radiotherapy and immune checkpoint blockade is an important factor for clinical translation. Future studies will systematically compare pre‐RT, concurrent, and post‐RT Mn@RM vaccination schedules to determine the optimal therapeutic sequence.

### 2.7 TCR Repertoire Analysis Reveals Mixed TAA Associated and Radiation Associated Antigen (RSA) Candidate Clonotype Expansion After Mn@RM Vaccination

**FIGURE 6:**
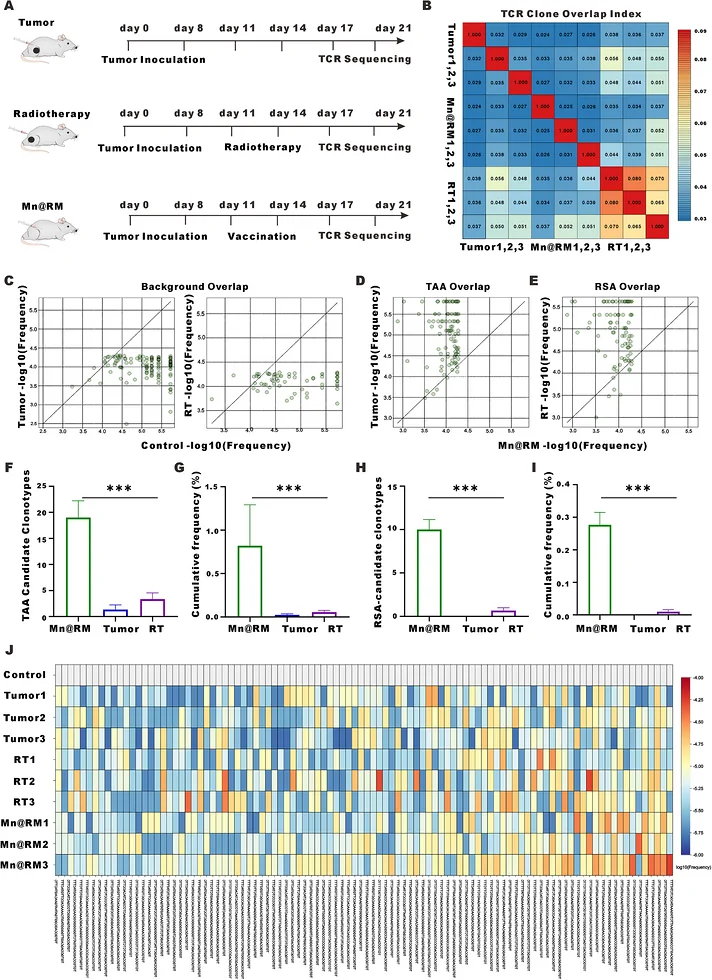
TCR repertoire analysis reveals mixed TAA‐associated and radiation associated candidate clonotype expansion after Mn@RM vaccination. (A) Schematic illustration of the experimental design for lymph node TCR sequencing. (n = 3) (B) Heatmap showing pairwise TCR clonotype overlap indices among Tumor, Mn@RM, and RT samples. Each square represents the overlap index between two individual samples. (C) Scatter plots showing background/public clonotype overlap between Control and Tumor or RT samples, clone frequencies are shown as −log10‐transformed values. (D) Scatter plot showing TAA‐candidate clonotype overlap between Mn@RM and Tumor samples. (E) Scatter plot showing RT/TSA‐candidate clonotype overlap between Mn@RM and RT samples. (F, G) Quantification of high‐frequency TAA‐candidate clonotypes, including the number of candidate clonotypes (F) and their cumulative clonotype frequency (G) in each group. (H, I) Quantification of high‐frequency RSA‐candidate clonotypes, including the number of candidate clonotypes (H) and their cumulative clonotype frequency (I) in each group. (J) Heatmap showing the distribution of representative candidate TCR clonotypes across Control, Tumor, RT, and Mn@RM samples. Data are presented as mean±SEM. Statistical significance was determined by one‐way ANOVA or unpaired two‐tailed Student's t‐test, as appropriate. ***p < 0.001.

To further determine whether Mn@RM vaccination induced antigen‐associated T cell clonal expansion, TCR sequencing was performed on lymph node T cells from Control, Tumor‐bearing, RT‐treated, and Mn@RM‐vaccinated mice (Figure 6A). To obtain an overview of treatment‐associated TCR repertoire remodeling, pairwise TCR clonotype overlap analysis was performed among Tumor, RT, and Mn@RM groups (Figure 6B). The heatmap showed that RT samples exhibited relatively evident overlap with both Tumor and Mn@RM samples, while intra‐group comparisons within the Mn@RM and Tumor groups also displayed a certain degree of clonotype overlap. The overlap between Mn@RM and Tumor samples suggests that Mn@RM retained conventional tumor‐associated antigen components from B16F10 tumor cells, whereas the overlap between Mn@RM and RT samples indicates that Mn@RM may partially recapitulate the antigenic features of irradiated tumors. Notably, the relatively higher intra‐group overlap among RT samples suggests that radiotherapy may induce a more convergent TCR repertoire remodeling, potentially associated with radiation‐remodeled antigenic features. The detectable overlap of Mn@RM with both Tumor and RT groups therefore supports the possibility that Mn@RM vaccination induces TCR expansion related to tumor‐associated antigens, and its closer overlap with RT further suggests its potential to expand RSA candidate clonotypes. However, the intra‐group overlap within Mn@RM and Tumor groups was not as uniform as that observed in the RT group, although a certain degree of similarity was still present. This may be attributed to background/public TCR repertoires shared among C57BL/6 mice, individual TCR heterogeneity, and the polyantigenic nature of Mn@RM and tumor‐derived antigen responses, in which different mice may preferentially expand TCR clones against different dominant epitopes. Therefore, to more specifically distinguish antigen‐associated clonotypes from background overlap, subsequent analyses focused on Control filtered TAA candidate and RSA candidate shared clonotypes and their cumulative clonotype frequencies.

To further distinguish antigen associated clonotype overlap from background/public TCR sharing, Control related clonotypes were first analyzed by comparing the Control mouse with Tumor and RT samples (Figure 6C). The detectable overlap between Control and tumor bearing or RT treated mice indicated that a fraction of shared clonotypes could arise from the intrinsic C57BL/6 TCR background or public clonotypes rather than treatment specific antigen responses. Therefore, Control detected clonotypes were excluded from subsequent candidate clonotype analyses to refine the identification of antigen associated TCR expansion. After this background filtering, Mn@RM still shared a subset of clonotypes with Tumor samples, supporting the presence of TAA candidate clonotypes derived from conventional B16F10 tumor associated antigens (Figure 6D). In parallel, Mn@RM also shared a distinct subset of clonotypes with RT samples after excluding both tumor and control associated backgrounds, suggesting the presence of radiation‐associated candidate clonotypes potentially linked to radiation‐remodeled antigenic features (Figure 6E). These results indicate that Mn@RM vaccination does not elicit a single source TCR response, but rather induces a broad antigen associated clonotype repertoire containing both conventional TAA related and radiation associated candidate components. This mixed clonotype pattern highlights the advantage of Mn@RM as a whole antigen tumor membrane vaccine, which can simultaneously preserve preexisting tumor associated antigens and radiation remodeled antigenic information without requiring prior identification of individual neoantigens.

To quantitatively evaluate Mn@RM‐induced antigen‐associated TCR clonal expansion, two candidate clonotype pools were defined after excluding Control detected background/public clonotypes. TAA candidate clonotypes were defined as clonotypes shared between Mn@RM and Tumor samples but absent from the Control mouse, whereas RSA candidate clonotypes were defined as clonotypes shared between Mn@RM and RT samples but absent from both Tumor and Control mice. High frequency candidate clonotypes were further defined as clonotypes with Clone (Fraction ≥ 0.0001), and the cumulative frequency of these clonotypes in each sample was calculated by summing the clone fraction values of all candidate clonotypes. Fraction values for all candidate clonotypes in each sample. Among the Control filtered TAA candidate clonotypes, Mn@RM vaccinated mice showed the highest number of high frequency clonotypes, with an average of 19.00 ± 3.21 clonotypes per mouse, compared with 1.33 ± 0.88 in the Tumor group, 3.33 ± 1.20 in the RT group (Figure 6F). Consistently, the cumulative frequency of high‐frequency TAA candidate clonotypes was markedly increased in the Mn@RM group, reaching 0.591 ± 0.245%, compared with 0.024 ± 0.013% in Tumor, 0.056 ± 0.020% in RT (Figure 6G). These data indicate that Mn@RM vaccination effectively enriched TAA associated candidate TCR clonotypes, suggesting that the irradiated tumor membrane vaccine preserved conventional tumor‐associated antigenic components and promoted their corresponding TCR clonal expansion. For RSA candidate clonotypes, Mn@RM vaccinated mice also displayed the highest degree of clonal enrichment. The average number of high‐frequency RSA candidate clonotypes was 10.00 ± 1.15 clonotypes per mouse in the Mn@RM group, compared with 0.67 ± 0.33 in the RT group (Figure 6H). Similarly, the cumulative frequency of high‐frequency RSA candidate clonotypes was 0.273 ± 0.038% in Mn@RM vaccinated mice, whereas it was only 0.008 ± 0.004% in RT mice and 0% in Tumor and Control mice (Figure 6I). These results suggest that Mn@RM not only induces TAA associated candidate clonotype expansion but also enriches a subset of RSA candidate clonotypes associated with radiation‐remodeled antigenic features. Together, the higher clonotype numbers and cumulative frequency contributions of both TAA and RSA candidate clonotypes support Mn@RM as a promising whole‐antigen tumor membrane vaccine capable of inducing broad tumor antigen associated TCR clonal responses. The representative clonotype heatmap showed that Mn@RM samples contained multiple candidate TCR clonotypes partially shared with Tumor or RT samples, while these clonotypes were largely absent in the Control sample (Figure 6J). This pattern further supports that Mn@RM vaccination induced a broad antigen‐associated TCR response rather than nonspecific background expansion. Together with the quantitative analyses of TAA candidate and RSA candidate clonotypes, these results indicate that Mn@RM can carry diverse tumor antigenic information and promote corresponding T cell clonal expansion, highlighting its potential as a promising whole antigen anti‐tumor vaccine platform.

## 3 Conclusions

In this study, a radiotherapy‐treated tumor membrane‐based nanovaccine, Mn@RM, was developed by integrating irradiated tumor cell membranes with manganese‐based metal‐organic framework nanoadjuvants. Whole‐exome sequencing and RNA‐seq analysis demonstrated that radiotherapy associated mutations could be partially recapitulated between in vitro irradiated tumor cells and in vivo irradiated tumor tissues in the B16‐F10 melanoma model. This observation was further supported in the Lewis lung carcinoma model, indicating that the reproducible detection of radiotherapy associated mutational events is not restricted to a single tumor cell line. These findings provide a genomic rationale for using RM as a broad antigen source that may contain tumor‐associated antigens, radiotherapy remodeled antigens, and potential radiotherapy induced neoantigenic components. Mn@RM retained the membrane associated antigenic repertoire of irradiated tumor cells and enabled the co‐delivery of tumor membrane antigens and Mn‐based immune adjuvants. Owing to its nanoscale structure, Mn@RM efficiently accumulated in lymph nodes after subcutaneous vaccination, thereby relocating antigen presentation from the immunosuppressive tumor microenvironment to immune cell rich lymphoid tissues. The Mn‐MOF nanoadjuvant further enhanced dendritic cell maturation, promoted antigen presentation, and activated immune‐related signaling pathways, including cGAS‐STING associated immune activation. Consistently, Mn@RM improved lymph node immune activation and promoted CD4^+^ and CD8^+^T cell infiltration and activation in the tumor microenvironment.

TCR repertoire analysis further showed that Mn@RM induced broad antigen associated T cell clonal expansion. The expanded clonotype repertoire contained both tumor associated antigen candidate clonotypes and radiation associated candidate clonotypes, supporting the concept that Mn@RM functions as a whole antigen tumor membrane vaccine. This broad clonal response is consistent with the antigenic complexity of radiotherapy‐treated tumor membranes and highlights the advantage of using intact tumor membrane antigens to amplify radiotherapy‐associated anti‐tumor immunity. Overall, this study demonstrates that RM can serve as an immunogenic and broadly representative antigen source for therapeutic vaccination, and that Mn‐MOF nanoadjuvants can further enhance antigen presentation and vaccine‐induced anti‐tumor immunity. Compared with peptide neoantigen vaccine strategies, this membrane based approach avoids the need for predefined selection and synthesis of individual neoantigen peptides, providing a practical platform for combining tumor membrane vaccination with radiotherapy and immune checkpoint blockade.

## 4 Limitation

In this study, although several candidate radiotherapy‐induced neoantigens were identified by WES/RNA‐seq analysis and NetMHCpan prediction, the immunogenicity and functional contribution of individual predicted peptides were not directly validated. Since Mn@RM is a whole radiotherapy‐treated tumor membrane‐based vaccine, its anti‐tumor efficacy is likely mediated by a broad antigenic repertoire that includes tumor‐associated antigens, radiotherapy remodeled antigens, and potential radiotherapy induced neoantigenic components, rather than by a single defined peptide. Therefore, this study mainly supports the feasibility of using radiotherapy‐treated tumor membranes as an immunogenic vaccine source for combination with radiotherapy. Future peptide‐specific ELISPOT assays, MHC‐I multimer staining, single cell TCR analysis, and functional blocking experiments will be needed to define the contribution of individual radiotherapy‐induced neoantigens.

This study provides proof‐of‐concept evidence that Mn@RM can amplify radiotherapy‐associated anti‐tumor immunity, several experiments were performed with relatively small sample sizes, particularly the mechanistic immune profiling and sequencing‐based analyses. While statistically significant differences were observed in key endpoints, larger cohorts will be required in future studies to further strengthen statistical power and validate therapeutic robustness. In addition, although the Lewis lung carcinoma model was included to support the applicability of the radiotherapy‐induced mutation screening strategy beyond B16‐F10 melanoma cells, further validation in additional tumor models will be important to assess the broader generalizability of this platform. Moreover, this study demonstrated that Mn@RM enhances dendritic cell maturation, antigen presentation, and T cell activation, the immunosuppressive tumor microenvironment was not comprehensively investigated. In particular, regulatory T cells, myeloid‐derived suppressor cells, tumor‐associated macrophages, and suppressive dendritic cell subsets were not systematically analyzed. Future studies will further characterize these immunosuppressive components and explore whether combining Mn@RM with strategies targeting the immunosuppressive microenvironment can further improve the therapeutic efficacy of radiotherapy‐based immunotherapy.

## 5 Materials and Methods

### 5.1 Chemicals and Materials

DiR was purchased from MedChemExpress (MCE); HEPES and the BCA assay kit were purchased from Thermo Fisher Scientific; 1,3,5‐Benzenetricarboxylic acid, sucrose, penicillin/streptomycin, Paraformaldehyde, MnCl_2_, and OVA were purchased from Sigma‐Aldrich (USA). Dulbecco's Modified Eagle's Medium (DMEM), Roswell Park Memorial Institute (RPMI)‐1640 medium and Fetal Bovine Serum (FBS) were purchased from Gibco Life Technologies (Grand Island, NY, USA). All flow antibodies were purchased from BioLegend (San Diego, CA, USA). Collagenase IV and hyaluronidase were purchased from Biosharp (Hefei, China), Anti–PD‐1 monoclonal antibody (clone RMP1‐14, rat IgG2a) for treatment was obtained from BioXcell (USA). The protease inhibitor cocktail was purchased from Roche (USA).

### 5.2 Cells and Animals

The B16 cell line was purchased from China Center for Type Culture Collection (Wuhan, China). All cells were cultured in an incubator at 37°C and 5% CO_2_ conditions. Male C57BL/6J mice were purchased from SHULAIBAO (Wuhan, China). Bone marrow–derived dendritic cells (BMDCs) were isolated from 6–8‐week‐old male C57BL/6 mice. Briefly, red blood cells were removed using RBC lysis buffer, and the remaining cells were cultured in RPMI‐1640 medium supplemented with 10% FBS and 20 ng/mL granulocyte–macrophage colony‐stimulating factor (GM‐CSF). The culture medium was replaced on day 3, and DCs were harvested on day 6 for experiments. All animal experiments were performed in accordance with the guidelines approved by the Laboratory Animal Care and Use Committee of Hainan Medical University.

### 5.3 Bioinformatics Analysis and Neoantigen Prediction

The expression of HLA‐A genes in tumor, metastasis and normal tissues were analyzed using the TIMER database (http://cistrome.shinyapps.io/timer/). RNA sequencing (RNA‐seq) and whole‐exome sequencing (WES) were performed by BGI Genomics (Shenzhen, China). Raw sequencing reads were filtered using SOAPnuke to remove low‐quality reads and obtain clean data. The clean reads were then mapped to the mouse reference genome (Mus musculus, GCF_000001635.26_GRCm38.p6) using the Burrows Wheeler Aligner (BWA). The aligned BAM files were further processed for duplicate removal using the Picard toolkit following GATK best‐practice guidelines (http://www.broadinstitute.org/gatk/guide/best‐practices). In addition, WES and RNA‐seq were performed on B16‐F10 tumors collected from C57BL/6 mice after radiotherapy. To further validate the general applicability of this workflow, untreated Lewis lung carcinoma cells, in vitro irradiated Lewis cells, and irradiated Lewis tumor tissues were analyzed using the same WES/RNA‐seq‐based mutation screening and NetMHCpan‐based MHC‐I epitope prediction strategy. For RNA‐seq analysis, gene expression levels were normalized using transcripts per million (TPM). The NetMHCpan v4.1 algorithm (https://services.healthtech.dtu.dk) was used to predict MHC‐I binding affinities of all possible 8–11‐mer mutation‐derived peptides identified after radiotherapy, with 200 nM used as the cutoff value for predicted binding affinity.

### 5.4 Preparation of RM, Mn‐MOFs and Mn@RM

To prepare RM, B16F10 cells were cultured in 10 cm cell culture dishes and irradiated with 6‐MV X‐rays at a dose of 20 Gy (CHIRAD 225). After 24 h, the irradiated cells were collected and suspended in a solution containing 100 mM Tris‐HCl, 0.5 mM EDTA, 70 mM sucrose, and a protease inhibitor cocktail. The suspension was subjected to ultrasonic treatment at 400 W for 5 min. The supernatant was first collected by centrifugation at 1200 × g for 10 min at 4°C, followed by further centrifugation at 12,000 × g for 60 min to purify the cancer cell membranes. Mn‐MOF nanoparticles were synthesized by coordination of MnCl_2_ with 1,3,5‐benzenetricarboxylic acid. Specifically, 90 µL of 0.2 M sodium 1,3,5‐benzenetricarboxylate and 90 µL of 0.3 M MnCl_2_ were added to a 10 mL mixed solution containing 0.5 M Hexanol, 0.05 M cetyltrimethylammonium bromide and 0.5 M isooctane. The mixture was vigorously stirred for 10 min at room temperature before being transferred to a hydrothermal reactor. The mixture was then heated to 120°C for 6 h. After cooling to room temperature, the reaction solution was centrifuged at 13 000 rpm for 10 min, and the supernatant was discarded, and then dispersed and washed with 5 mL of ethanol. The ethanol suspension was centrifuged at 13 000 rpm for 10 min, and this step was repeated three times before being dried in a vacuum drying oven to obtain Mn‐MOF nanoparticles. Mn@RM was prepared by mixing the RM and Mn‐MOFs nanoparticles in a 1:1 (w/w) ratio. The mixture was vortexed for 5 min and gently stirred for 2 h at 4°C. Finally, the Mn@RM nanovaccine was obtained by extruding the mixture through polycarbonate membranes with pore sizes of 1000 and 400 nm using a liposome extruder. The protein concentration of Mn@RM was determined using a bicinchoninic acid (BCA) assay kit.

### 5.5 Characterization of Mn‐MOFs and Mn@RM

The structures of Mn‐MOFs and Mn@RM were characterized by transmission electron microscopy (TEM, Tecnai G20, FEI Corp, USA). The surface morphology and distribution of Mn, C, O, P elemental mapping images in Mn@RM and Mn‐MOFs were observed by Field Emission Scanning Electron Microscope (FESEM) equipped with energy‐dispersive X‐ray spectroscopy (EDS). The surface Zeta potential and hydrodynamic diameter of Mn‐MOFs and Mn@RM were measured using a Zetasizer Nano ZS (Malvern Instruments Ltd., Malvern, UK).

### 5.6 In Vitro DCs Uptake, Maturation and Antigen Presenting Function Assays

To identify the time required for Mn@RM uptake by BMDCs, DiR (Excitation wavelength:748 nm, Emission wavelength:780 nm) labeled Mn@RMs (5 µg/mL) were co‐culture with BMDCs for 2, 6, 12 and 24 h. To verify the effect of radiotherapy on cell surface MHC I expression, B16 cells (1 × 10^7^) were seeded in 10 cm culture dishes and irradiated with 20 Gy using 6‐MV X‐rays (CHIRAD 225). At 8, 12, 24, and 32 h after irradiation, the treated cells were collected and analyzed by flow cytometry (CytoFLEX S, Beckman Coulter, USA). For BMDCs maturation assay, PBS, RM and Mn@RM (BCA concentration: 10 µg/mL) were co‐cultured with BMDCs. After 24 h, BMDCs were collected and washed with FACS buffer, and stained on ice with the following antibodies: CD11c (APC, 1:200 dilution; BioLegend), CD40 (PE, 1:200 dilution; BioLegend), CD80 (FITC, 1:200 dilution; BioLegend), CD86 (PerCP‐Cy5.5, 1:200 dilution; BioLegend) and MHC II (BV510, 1:200 dilution; BioLegend) flow antibody.For the BMDC antigen presentation assay, BMDCs were incubated with OVA protein (1 µg/mL), OVA‐B16‐RM and OVA‐B16‐Mn@RM (10 µg/mL). After co‐incubation, BMDCs were collected and washed with FACS buffer and then stained on ice with CD11c and SIINFEKL/H‐2K^b^ monoclonal antibody.

### 5.7 In Vivo Distribution and Biosafety Analysis

To analyze the in vivo biodistribution of Mn@RM, DiR‐labeled Mn@RMs (10 mg/kg) were subcutaneously injected into healthy C57BL/6 mice. After 1, 4 and 12 h, mice were sacrificed and the lymph node, heart, spleen, lung, liver and kidney were collected and imaged using IVIS spectral imaging system. To analyze the biosafety of Mn@RM and Mn@RM (10 mg/kg) were subcutaneously injected into healthy C57BL/6 mice, after 21 days, the mice were sacrificed and the main organs were sectioned and stained with H&E.

### 5.8 In Vivo Lymph Node Immune Microenvironment Analysis

To analyze the effects of different tumor vaccines on immune cell phenotypes in lymph nodes, C57BL/6 mice were subcutaneously injected with PBS, RM and Mn@RM (BCA concentration:3 mg/kg) at day 0, 2, 7 and 11. On day 14, the mice were sacrificed and lymph nodes were collected and prepared as single‐cell suspensions. Then, the cells were stained with the following antibodies: CD11c (APC, 1:200 dilution; BioLegend), CD80 (PE, 1:200 dilution; BioLegend), CD86 (FITC, 1:200 dilution; BioLegend), CD3 (APC, 1:200 dilution; BioLegend), CD4 (FITC, 1:200 dilution; BioLegend), CD8 (PE, 1:200 dilution; BioLegend) and CD69 (BV510, 1:200 dilution; BioLegend) and analyzed by flow cytometry to evaluate DC maturation and T cell activation.

### 5.9 Ex Vivo T Cell Re‐Stimulation Assay

To further evaluate whether vaccination induced T cells responsive to radiotherapy treated tumor membrane‐associated antigens, mice were subcutaneously vaccinated with PBS, CM, RM, or Mn@RM. Fourteen days after vaccination, spleens were collected under sterile conditions and mechanically dissociated into single‐cell suspensions. Red blood cells were removed using RBC lysis buffer, and the remaining splenocytes were washed and resuspended in complete RPMI‐1640 medium. For ex vivo antigen re‐stimulation, splenocytes from each group were co‐cultured with RM as the antigenic stimulus. After co‐culture, cells were harvested and stained with flow cytometry antibodies against CD3, CD8, and CD69. CD8^+^ T cell activation was evaluated based on the proportion of CD8^+^CD69^+^ T cells, the cells were filtered and measured by flow cytometry.

### 5.10 In Vivo Cancer Treatment

To explore the synergistic anti‐tumor effect of radiotherapy and Mn@RM vaccine, male C57BL/6 mice (4–6 weeks old) were divided into four groups (PBS, RT, Mn@RM and Mn@RM + RT, n = 5). B16F10 cells (2 × 10^6^) were subcutaneously injected into the right flank of the mice. Mn@RM (10 mg/kg) were subcutaneously injected at day 8, day 10, 13, 16 and 19, Radiotherapy (3 × 8 Gy) treatment was administered on day 8, 11, 14 and 17. Tumor growth and body weight were monitored every other day. To explore the synergistic anti‐tumor effect of radiotherapy, male C57BL/6 mice (4–6 weeks old) were divided into four groups (PBS, RT + PD‐1, Mn@RM + RT and Mn@RM + RT + PD‐1, n = 5). 2 × 10^6^ B16F10 cells were subcutaneously injected into the right flank of the mice. Mn@RM (10 mg/kg) were subcutaneously injected at day 8, 10, 13, 16 and 19; Radiotherapy (3 × 8 Gy) treatment were administered at day 8, day 11, day 14 and day 17. Anti‐PD‐1 antibody (10 mg/kg) was intraperitoneally injected at day 9, 12, 15 and 18. Tumor growth and body weight were monitored every other day, and tumor volume was calculated as: volume = length × width^2^ × 0.52.

### 5.11 In Vivo Tumor Microenvironment Assay

Briefly, tumors were collected, cut into small pieces, and incubated with collagenase IV (0.32 mg mL^−1^) and hyaluronidase (0.5 mg mL^−1^) for 1 h at 37°C. The tumors were then digested into single‐cell suspensions and treated with red blood cell (RBC) lysis buffer. Then filtered and stained with CD3 (APC, 1:200 dilution; BioLegend), CD4 (FITC, 1:200 dilution; BioLegend), CD8 (BV421, 1:200 dilution; BioLegend), CD69 (BV510, 1:200 dilution; BioLegend) and PD‐L1 (PE, 1:200 dilution; BioLegend) flow antibody. After staining at 4°C for 30 min, the cells were filtered and measured by flow cytometry.

### 5.12 TCR Sequencing and Clonotype Analysis

To evaluate antigen associated T cell clonal expansion after Mn@RM vaccination, draining lymph nodes were collected from Control, Tumor‐bearing, RT treated, and Mn@RM vaccinated mice for TCR repertoire sequencing. TCR clonotypes were defined by the combination of CDR3 amino acid sequence, V segment, and J segment. Clone Fraction was calculated as the proportion of the read count of each clonotype relative to the total read count of all clonotypes within the same sample. High‐frequency clonotypes were defined as clonotypes with Clone. Fraction ≥ 0.0001. Pairwise clonotype overlap analysis was performed to evaluate global TCR repertoire similarity among Tumor, RT, and Mn@RM groups. To distinguish antigen associated clonotype expansion from background/public TCR sharing, clonotypes detected in the Control mouse were excluded from subsequent candidate clonotype analyses. TAA candidate clonotypes were defined as clonotypes shared between Mn@RM vaccinated and tumor bearing mice but absent from the Control mouse. Radiation associated antigen RSA candidate clonotypes were defined as clonotypes shared between Mn@RM vaccinated and RT treated mice but absent from both tumor bearing and control mice. Cumulative clonotype frequency was calculated as the sum of Clone fraction values for all candidate clonotypes within each sample, and representative candidate clonotypes were visualized by heatmap analysis using ‐log10 transformed clone frequency values.

### 5.13 Statistical Analysis

All experimental data were analyzed by GraphPad Prism 8.0 software and presented as mean ± s.d. or s.e.m. Two‐tailed t‐tests and one‐way analysis of variance (ANOVA) were performed to two, three or more groups. Significant differences between two groups were indicated by *p < 0.05, **p < 0.01, ***p < 0.001, and ****p < 0.0001.

## Author Contributions

**Yiyu Wang**, **Qiqi Qi** and **Dezhong Li** contributed equally to this work. In this work, all authors contributed to the preparation of the manuscript and figures. Each author reviewed figures and approved the final version of the manuscript.

## Ethics Approval and Consent to Participate

All animal experiments were carried out after approval by the Ethical Committee for Experimental Animal Care and Use of Hainan Medical University (approval number: HYALL‐2025‐314) and according to the policy of the National Ministry of Health of China.

## Conflicts of Interest

The authors declare no conflicts of interest.

## Supporting information




**Supporting File**: advs75860‐sup‐0001‐SuppMat.docx.

## Data Availability

The data that support the findings of this study are available from the corresponding author upon reasonable request.
