# Supplementary material for: Combining Radiation‐Treated Tumor Vaccines With Mn‐MOF Nanoadjuvants to Amplify Radiation Induced Anti‐Tumor Immune Responses
Source: Adv Sci (Weinh). 2026 May 29:e75860. Online ahead of print. doi: 10.1002/advs.75860 (PMC13336090; doi:10.1002/advs.75860)
Supplement: Supplementary file 1 — Supporting File: advs75860‐sup‐0001‐SuppMat.docx. [file ADVS-9999-e75860-s001.docx]

**Combining Radiation-Treated Tumor Vaccines with Mn-MOF Nanoadjuvants to Amplify Radiation Induced Anti-Tumor Immune Responses**

Yiyu Wang^1^, Qiqi Qi^5^, Dezhong Li^1 2^, Jingshi Tang^1 2^, Weifeng Wang^4^, Zushun Xu^5^, Qi Xu^3^, Fangfang Du^1 2^ Qianyuan He^1 2*^

^1^NHC Key Laboratory of Tropical Disease Control, Engineering Research Center for Hainan Bio-Smart Materials and Bio-Medical Devices, Key Laboratory of Hainan Functional Materials and Molecular Imaging, School of Life Sciences and Medical Technology, Hainan Medical University, Haikou, Hainan, 571199, China

^2^Key Laboratory of Emergency and Trauma, Ministry of Education, Key Laboratory of Haikou Trauma, Key Laboratory of Hainan Trauma and Disaster Rescue, The First Affiliated Hospital, Hainan Medical University, Haikou 571199, China

^3^College of Life Sciences, Yangtze University, Jingzhou 434025, China

^4^Hainan Cancer Hospital, Affiliated Hospital of Hainan Medical University, Haikou, Hainan, China；

^5^School of Materials Science and Engineering, Hubei University, Wuhan 430062, China

Figure S1


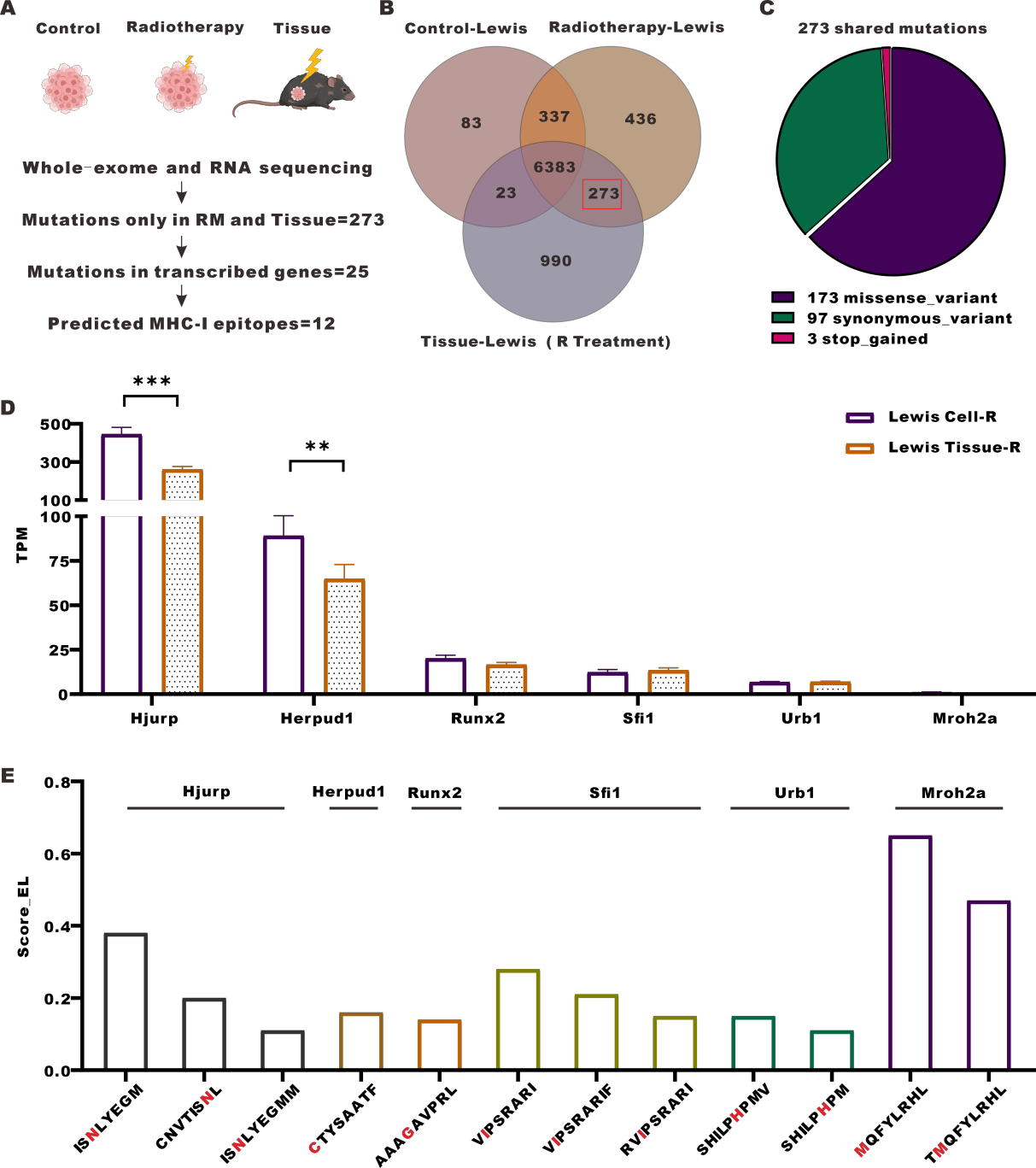


Figure S1. Validation of radiotherapy-induced mutation-derived neoantigen prediction in Lewis lung carcinoma cells. (A) Schematic workflow for identifying radiotherapy induced mutation derived candidate neoantigens in the Lewis lung carcinoma model. Whole exome sequencing and RNA sequencing were performed on untreated Lewis cells, in vitro irradiated Lewis cells, and irradiated Lewis tumor tissues. Mutations shared between irradiated Lewis cells and irradiated Lewis tumor tissues were further filtered by gene expression and MHC-I binding prediction. (B) Venn diagram showing the overlap of somatic mutations among untreated Lewis cells, in vitro irradiated Lewis cells, and irradiated Lewis tumor tissues. (C) Classification of the 273 shared mutations by mutation type, including missense variants, synonymous variants, and stop-gained mutations. (D) RNA expression levels of selected genes carrying nonsynonymous shared mutations in irradiated Lewis cells and irradiated Lewis tumor tissues. (E) Predicted MHC-I binding scores of mutation containing 8-11 mer peptides derived from expressed shared mutant genes, mutation sites are highlighted in red (n=4). All data are presented as the mean ± SEM. P-values of the experiments were calculated by one-way ANOVA. *P<0.05, **P<0.01, ***P<0.001.

Figure S2:


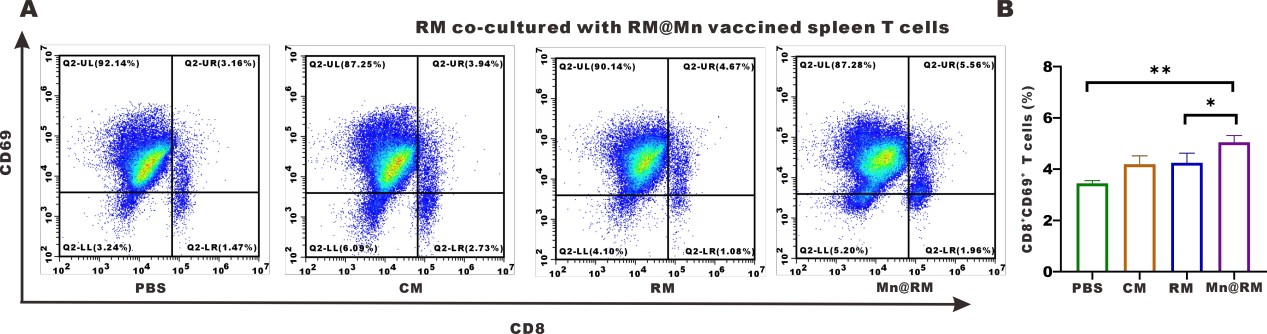


Figure S2. Flow cytometry analysis of CD8^+^T cell activation following vaccination with PBS, CM, RM and Mn@RM. (n=5) (A) Representative flow cytometry plots of CD69 expression on CD8^+^T cells after co-culturing splenocytes from PBS, CM, RM, or Mn@RM vaccinated mice with RM. (B) The proportion of CD8^+^CD69^+^T cells. All data are presented as the mean±SEM. P-values of the experiments were calculated by one-way ANOVA. *P<0.05, **P<0.01, ***P<0.001.
